# Characterization of *Pasteurella multocida* isolated from dead rabbits with respiratory disease in Fujian, China

**DOI:** 10.1186/s12917-019-2191-3

**Published:** 2019-12-04

**Authors:** Jinxiang Wang, Lei Sang, Shikun Sun, Yanfeng Chen, Dongjin Chen, Xiping Xie

**Affiliations:** Institute of Animal Husbandry and Veterinary Medicine, Fujian Academy of Agricultural Sciences, No. 100 Pudang Road, Xindian Town, Jin’an District, Fuzhou, Fujian Province People’s Republic of China

**Keywords:** *Pasteurella multocida*, Rabbit, Serotype, Multi-locus sequence typing, Virulence factors, Antimicrobial susceptibility

## Abstract

**Background:**

*Pasteurella multocida* is one of the important pathogens that infect rabbits, causing major economic losses in commercial rabbit farming. In this study, 205 *P. multocida* isolates recovered from lungs of dead rabbits with respiratory disease were defined by capsular serogroups, lipopolysaccharide (LPS) genotypes, multi-locus sequence types and screened virulence factors by using PCR assays, and tested antimicrobial susceptibility.

**Results:**

The 205 isolates were assigned into 2 capsular types, A and D, and 2 LPS genotypes, L3 and L6. When combining capsular types with LPS genotypes, 4 serotypes were detected. A:L3 (51.22%, 105/205) was the most predominant serotype, followed by A:L6 (24.88%, 51/205), D:L6 (19.02%, 39/205) and D:L3 (4.88%, 10/205). The 205 isolates were grouped into 3 sequence types, ST10, ST11 and ST12. ST12 (56.10%, 115/205) was the most prevalent sequence type, followed by ST10 (24.88%, 51/205) and ST11 (19.02%, 39/205). In the 205 isolates, virulence associated genes *ptfA*, *fur*, *hgbB*, *ompA*, *ompH* and *oma87* were positive in the PCR screening, whereas the *toxA* and *tbpA* genes were negative. Notably, the 156 capsular serogroup A isolates carried the *pmHAS* gene. All the 205 isolates were susceptible to most of the used antibiotics, except for streptomycin, gentamycin, kanamycin and ceftriaxone, and the resistance rates of which were 27.80, 15.61, 9.27 and 2.44%, respectively.

**Conclusions:**

This study, for the first time, described the prevalence and characteristics of *P. multocida* causing respiratory disease in rabbits in Fujian Province, which might be useful for tracking the epidemic strains and development of efficient vaccines and methods to prevent and control the pathogen.

## Background

*Pasteurella multocida* is considered as one of the important pathogens that infects a wide range of animals [1]. The infection of *P. multocida* is associated with economically important diseases, including porcine progressive atrophic rhinitis, porcine respiratory disease complex, bovine haemorrhagic speticaemia, avian cholera and rabbit respiratory disease [1, 2]. In recent years, the *P. multocida* strains from different animal species were characterized to understand the epidemiology and pathogenic mechanisms of the organism [3–6].

*P. multocida* is recognized as an endemic pathogen in rabbits, which was reported in different countries and regions [5–10]. The *P. multocida* infected rabbits are mainly characterized by rhinitis, tracheitis, pneumonia and otitis media [1, 6, 7]. In Fujian Province, in the southeast of China, rabbit farms are mainly located in the 3 cities of Fuzhou, Longyan and Nanping, and about 60% of rabbit farms are located in Longyan. Four rabbit breeds are raised in Fujian Province, including 3 local rabbit breeds and a foreign rabbit breed. The Fujian Yellow rabbit (local rabbit breed) is raised in Fuzhou, the Fujian White rabbit and Minxinan Black rabbit (local rabbit breeds) are raised in Longyan, and the Hyplus rabbit (foreign rabbit breed) is raised in Nanping. To our knowledge, pasteurellosis was common and widespread in rabbits in Fujian Province, whereas the knowledge about the epidemiology and characterization of *P. multocida* was limited. In this study, *P. multocida* strains were isolated from lungs of dead rabbits with respiratory disease and the isolates were then defined by capsular types, LPS genotypes and multi-locus sequence types, screened virulence factors and antimicrobial susceptibility.

## Results

### *P. multocida* isolation and identification

A total of 205 strains were recovered from the 530 lung samples. The amplified 1549 bp *16S rRNA* genes of the 205 isolates shared the highest identity (ranged from 99 to 100%) with that of *P. multocida*, suggesting that these isolates were *P. multocida* strains.

### Capsular and LPS typing

The 205 *P. multocida* isolates were classified into 2 capsular types (A and D) and 2 LPS genotypes (L3 and L6). When combining capsular types with LPS genotypes, A:L3 was the most prevalent serotype (51.22%, 105/205), followed by A:L6 (24.88%, 51/205), D:L6 (19.02%, 39/205) and D:L3 (4.88%, 10/205) (Table 1). Eight out of 9 rabbit farms were co-infected by the *P. multocida* strains belonging to at least 2 serotypes (Table 1).

**Table 1:** The distribution, origin, capsular types, LPS genotypes and sequence types of the 205 *P. multocida* isolates

Cities	Rabbit farms	No. of samples	No. of isolates	Capsular types	LPS genotypes	STs
Fuzhou	Farm A	123	7	A	L6	10
			3	D	L6	11
			20	A	L3	12
			8	D	L3	12
	Farm B	98	21	A	L6	10
			12	D	L6	11
			18	A	L3	12
			1	D	L3	12
Longyan	Farm C	43	4	A	L6	10
			8	D	L6	11
			4	A	L3	12
	Farm D	58	2	D	L6	11
			25	A	L3	12
	Farm E	56	3	A	L6	10
			10	D	L6	11
			5	A	L3	12
			1	D	L3	12
	Farm F	30	2	A	L6	10
			1	D	L6	11
			7	A	L3	12
	Farm G	33	1	A	L6	10
			1	D	L6	11
			11	A	L3	12
Nanping	Farm H	56	13	A	L6	10
			2	D	L6	11
			8	A	L3	12
	Farm I	33	7	A	L3	12
Total		530	205			

### Multi-locus sequence typing

The MLST analysis revealed that the 205 *P. multocida* isolates were typed into 3 STs, ST10 (24.88%, 51/205), ST11 (19.02%, 39/205) and ST12 (56.10%, 115/205) (Table 1). The 51 isolates of serotype A:L6 belonged to ST10, the 39 isolates of serotype D:L3 belonged to ST11, and 105 isolates of serotype A:L3 and 10 isolates of serotype D:L3 belonged to ST12.

### Virulence associated genes detection

Among the 205 *P. multocida* isolates, the prevalence of 5 virulence associated genes of *ptfA*, *fur*, *hgbB*, *ompA*, *ompH* and *oma87* were 100%, whereas the prevalence of *toxA* and *tbpA* were 0%. The other 4 virulence genes of *tadD*, *pfhA*, *nanB* and *pmHAS* were also detected, and the prevalence rates were 82.44% (169/205), 37.07% (76/205), 48.78% (100/205) and 76.10% (156/205), respectively (Table 2).

**Table 2:** Prevalence of virulence genes among the 205 *P. multocida* isolates ordered by serotype

Serotypes	No. of isolates	Virulence genes											
		*ptfA*	*tadD*	*pfhA*	*toxA*	*fur*	*tbpA*	*hgbB*	*nanB*	*pmHAS*	*ompA*	*ompH*	*oma87*
A:L6	51	51	51	0	0	51	0	51	51	51	51	51	51
D:L6	39	39	13	5	0	39	0	39	39	0	39	39	39
A:L3	105	105	105	71	0	105	0	105	0	105	105	105	105
D:L3	10	10	0	0	0	10	0	10	10	0	10	10	10
Total	205	205	169	76	0	205	0	205	100	156	205	205	205

### Antimicrobial susceptibility test

The results of antimicrobial susceptibility test showed that the 205 *P. multocida* isolates were susceptible or intermediate susceptible to the most of antibiotics tested. However, resistance to streptomycin, gentamycin, kanamycin and ceftriaxone were observed, and the resistance rates were 27.80, 15.61, 9.27 and 2.44% (Table 3). Moreover, no isolate resistant to ≥3 antibiotics was detected.

**Table 3:** Antimicrobial susceptibility test of the 205 *P. multocida* isolates

Antibiotics	No. of isolates			Percentage of resistance or sensitive	
	R	I	S	Resistance rate (%)	Sensitive rate (%)
Penicillin	0	0	205	0	100
Kanamycin	19	131	55	9.27	26.89
Ceftriaxone	5	23	177	2.44	86.34
Enrofloxacin	0	0	205	0	100
Levofloxacin	0	32	173	0	84.39
Cefminox	0	61	144	0	70.24
Streptomycin	57	95	53	27.80	25.85
Florfenicol	0	0	205	0	100
Ceftizoxime	0	0	205	0	100
Ciprofloxacin	0	0	205	0	100
Gentamycin	32	43	130	15.61	63.41
Azithromycin	0	18	187	0	91.22

“*R*” represents resistance, “*I*” represents intermediate, “*S*” represents susceptible

## Discussion

This study was the first to demonstrate the prevalence and characteristics of *P. multocida* from rabbits in Fujian Province, in the southeast of China. The results showed that infection of *P. multocida* was common in the 9 rabbitries of Fuzhou, Longyan and Nanping, and that co-infections of *P. multocida* strains of different serotypes were also detected. The prevalence rates of *P. multocida* in the lung samples of dead rabbits with respiratory disease ranged from 21.21 to 53.06%, suggesting that *P. multocida* was probable an important pathogen causing high mortality in the 9 rabbit farms in Fujian Province.

*P. multocida* strains were classified into 5 capsular types (A, B, D, E and F) and 8 LPS genotypes (L1-L8) [11, 12]. The 205 *P. multocida* isolates in this study were grouped into 4 serotypes of A:L6, A:L3, D:L6 and D:L3. In consistence with the previous reports, the isolates of capsular type A (76.10%) or LPS genotype L3 (56.10%) were the most predominate strains [6–10]. However, the highly pathogenic strain of capsular type F was not detected in this study [6, 7, 10, 13]. By now, the vaccines against *P. multocida* in rabbits in China were derived from the capsular type A strains, including C51–17 strain and JN strain. Thus it can be concluded that the vaccines do not fully match the epidemic strains.

By using the *P. multocida* Multi-host MLST scheme, the 205 *P. multocida* isolates in this study were clustered into 3 STs, ST10, ST11 and ST12, and the ST12 was the most prevalent sequence type (56.10%, 115/205). In the Multi-host MLST database, *P. multocida* strains isolated from rabbits belong to 9 STs, including ST10, ST11, ST12, ST25, ST35, ST72, ST73, ST76 and ST77. *P. multocida* strains belong to ST10, ST11 and ST12 are found in rabbits from Spain, strains belong to ST11 and ST12 are found in rabbits from Portugal, and strains belonged to ST12 are found in rabbits from China. A recent study based on the alternative RIRDC *P. multocida* MLST scheme showed that the 39 *P. multocida* strains isolated from rabbits in Italy belonged to 19 STs, and the ST50 strains might probably correspond to the ST11 strains in the Multi-host MLST database [6].

It is well recognized that the 12 virulence factors screened for in this study contributed to the pathogenicity of *P. multocida*. The results revealed that, with the exception of *toxA* and *tbpA* genes, the 10 other virulence genes showed high prevalence rates ranging from 37.07 to 100%. The *toxA* and *tbpA* genes were absent in the all 205 isolates. It is well recognized that the virulence associated gene *toxA* is associated with progressive atrophic rhinitis in swine [14], and this gene was not commonly found in *P. multocida* strains isolated from rabbits [6, 7, 15]. Garcia-Alvarez et al. first reported the presence of the *tbpA* gene in a *P. multocida* strain isolated from rabbit [7]. However, the *tbpA* gene was negative in this and other 2 studies [6, 15], suggesting that this gene might not essential for infection in rabbit. A previous report showed that the *hgbB* gene in the *P. multocida* strains of capsular type D belonged to ST11 was significantly associated with the respiratory disease in rabbits [7]. However, other studies showed that there were no relationships between *hgbB* gene and pasteurellosis in swine and sheep [16, 17]. Interestingly, an association between *pmHAS* and capsular type A was observed, since the gene was only detected in *P. multocida* strains belonged to capsular type A in this study. The *pmHAS* (hyaluronan synthase) promotes the formation of hyaluronic acid, the major composition of the capsular of capsular type A *P. multocida* strain [18].

Antibiotics are still the first choice for prevention and control the infections of *P. multocida* [19]. However, the imprudent use of antibiotics promoted the development of drug-resistant strains [8, 19, 20]. Although strains resistant to streptomycin, gentamycin, kanamycin and ceftriaxone were detected in the present study, no multidrug-resistant isolate was detected.

## Conclusions

The genetic diversity of the *P. multocida* strains in rabbits in Fujian Province was investigated in this study. Further efforts are needed to elucidate the pathogenic mechanisms of epidemic *P. multocida* strains of different capsular serogroups and LPS genotypes in rabbits, which will help in the selection of vaccines to control the pathogen [21, 22].

## Methods

### Ethics statement

The present study was approved by the Research Ethics Committee of the Institute of Animal Husbandry and Veterinary Medicine, Fujian Academy of Agriculture Sciences (Approved Number: FAAS-AHVM2017–0205).

### Sample collection and *P. multocida* isolation

From August 2017 to March 2019, 530 lung samples were collected from dead rabbits with respiratory disease from 9 rabbit farms in 3 cities (Fuzhou, Longyan and Nanping) of Fujian Province. Each lung sample was placed in a sterile tube, kept on ice and used for the isolation of *P. multocida* within 24 h. Each sample was homogenized to make 50% suspension in sterile phosphate buffered saline (PBS). One hundred microliter of each suspension was evenly plated on brain heart infusion (BHI) agar plate containing 5% sheep blood, and cultivated at 37 °C for 24 to 48 h. The suspected *P. multocida* colonies were selected and the *16S rRNA* genes of the suspected colonies were amplified [23]. The identities of the isolates were confirmed by sequencing the *16S rRNA* genes. The isolates selected for further characterization were each from a separate sample.

### Capsular and LPS typing

The capsular types and LPS genotypes of the *P. multocida* isolates were defined using multiplex PCR assays as reported previously [11, 12]. To confirm the identities of the fragments, the products from the 2 multiplex PCR assays were purified and sequenced. The serotype of the isolate was designated according to the combination of the capsular type with LPS genotype, such as A:L3 (capsular type A and LPS genotype L3).

### Multi-locus sequence typing

The isolates were analyzed by multi-locus sequence typing (MLST) using the *P. multocida* Multi-host MLST scheme (https://pubmlst.org/pmultocida/). Briefly, seven housekeeping genes (*adk*, *aroA*, *deoD*, *gdhA*, *g6pd*, *mdh* and *pgi*) of the *P. multocida* isolates were amplified by using PCR assays. Each 50 μL amplification reaction mixture comprised 25 μL 2 × *EasyTaq* PCR SuperMix (TransGen Biotech, Beijing, China), 4 μL of each forward and reverse primer (25 pmol/μL) and 50 ng chromosomal DNA. The PCR assays were carried out by 35 cycles of 94 °C for 30 s, 58 °C for 30 s, and 72 °C for 1 min, followed by a final extension step of 72 °C for 10 min. The expected PCR products were purified and then sequenced.

The allelic numbers of the seven housekeeping genes were assigned by comparing the sequences to the known allele sequences in the database. The sequence types (STs) of the isolates were defined according to the seven allelic numbers.

### Virulence associated genes detection

Twelve virulence genes were screened by PCR assays as previously reported (Table 4) [15, 24], including adhesion related proteins (*ptfA*, *tadD* and *pfhA*), dermonecrotoxin (*toxA*), iron binding proteins (*fur*, *tbpA* and *hgbB*), sialidases (*nanB*), hyaluronidase (*pmHAS*) and outer membrane proteins (*ompA*, *ompH* and *oma87*).

**Table 4:** Primers used for *16S rRNA* and 12 virulence genes

Genes	Primer sequence (5′-3′)	Product size (bp)	Reference
*16S rRNA*	F: ccgaattcgtcgacaacagagtttgatcctggctcag	1549	23
	R: cccgggatccaagcttaaggaggtgatccagcc		
*ptfA*	F: tgtggaattcagcattttagtgtgtc	488	24
	R: tcatgaattcttatgcgcaaaatcctgctgg		
*tadD*	F: tctacccattctcagcaaggc	416	24
	R: atcatttcgggcattcacc		
*pfhA*	F: ttcagagggatcaatcttcg	286	24
	R: aactccagttggtttgtcg		
*toxA*	F: cttagatgagcgacaagg	864	24
	R: gaatgccacacctctatag		
*fur*	F: gtttaccgtgtattagacca	244	24
	R: cattactacatttgccatac		
*tbpA*	F: ttggttggaaacggtaaagc	729	15
	R: taacgtgtacggaaaagccc		
*hgbB*	F: accgcgttggaattatgattg	789	15
	R: cattgagtacggcttgacat		
*nanB*	F: cattgcacctaacacctct	555	24
	R: ggacactgattgccctgaa		
*pmHAS*	F: tcaatgtttgcgatagtccgttag	430	24
	R: tggcgaatgatcggtgataga		
*ompA*	F: cgcatagcactcaagtttctcc	201	24
	R: cataaacagattgaccgaaacg		
*ompH*	F: cgcgtatgaaggtttaggt	438	24
	R: tttagattgtgcgtagtcaac		
*oma87*	F: ggcagcgagcaacagataacg	838	24
	R: tgttcgtcaaatgtcgggtga		

### Antimicrobial susceptibility test

The antimicrobial susceptibility of the isolates to 12 antibiotics was evaluated using disk diffusion method on BHI agar containing 5% sheep blood according to the Clinical and Laboratory Standards Institute (CLSI) standards [25]. The 12 antibiotics tested were penicillin, kanamycin, ceftriaxone, enrofloxacin, levofloxacin, cefminox, streptomycin, florfenicol, ceftizoxime, ciprofloxacin, gentamycin and azithromycin. The *Staphylococcus aureus* ATCC 29213 was used as a quality control. The breakpoints of the 12 antibiotics were determined according to CLSI interpretive standards.

## Data Availability

All data generated or analysed during this study are included in this published article.

## Abbreviations

BHI: Brain heart infusion
bp: base pair
LPS: Lipopolysaccharide
MLST: Multi-locus sequence typing
*P. multocida*: *Pasteurella multocida*
PCR: Polymerase chain reaction
STs: Sequence types
